# Mucosal immunization with Ad5-based vaccines protects Syrian hamsters from challenge with omicron and delta variants of SARS-CoV-2

**DOI:** 10.3389/fimmu.2023.1086035

**Published:** 2023-02-22

**Authors:** Molly R. Braun, Clarissa I. Martinez, Emery G. Dora, Laura J. Showalter, Annette R. Mercedes, Sean N. Tucker

**Affiliations:** Research & Development, Vaxart, Inc., South San Francisco, CA, United States

**Keywords:** SARS-CoV-2, omicron, delta, Syrian hamster, adenovirus type 5 (Ad5), variant of concern (VOC), challenge model

## Abstract

SARS-CoV-2 variant clades continue to circumvent antibody responses elicited by vaccination or infection. Current parenteral vaccination strategies reduce illness and hospitalization, yet do not significantly protect against infection by the more recent variants. It is thought that mucosal vaccination strategies may better protect against infection by inducing immunity at the sites of infection, blocking viral transmission more effectively, and significantly inhibiting the evolution of new variants of concern (VOCs). In this study, we evaluated the immunogenicity and efficacy of a mucosally-delivered, non-replicating, adenovirus type 5-vectored vaccine that expresses the spike (S) gene of Wuhan (rAd5-S-Wuhan), delta (rAd5-S-delta), or omicron (rAd5-S-omicron) SARS-CoV-2 VOCs. Hamsters were immunized with these vaccines intranasally prior to challenge with omicron or delta variants. Additionally, one group was vaccinated by oral gavage with rAd5-S-Wuhan prior to challenge with the delta variant. Both intranasal and oral administration of rAd5-S-Wuhan generated cross-reactive serum IgG and mucosal IgA to all variant spike and RBD proteins tested. rAd5-S-omicron and rAd5-S-delta additionally elicited cross-reactive antibodies, though rAd5-S-omicron had significantly lower binding antibody levels except against its matched antigens. Two weeks after the final vaccination, hamsters were challenged with a SARS-CoV-2 variant; omicron or delta. Whether matched to the challenge or with rAd5-S-Wuhan, all vaccines protected hamsters from weight loss and lung pathology caused by challenge and significantly reduced viral shedding compared to placebo. Vaccination with rAd5-S-Wuhan provided significant protection, although there was an improved reduction in shedding and disease pathology in groups protected by the matched VOC vaccines. Nevertheless, Wuhan-based vaccination elicited the most cross-reactive antibody responses generally. Overall, heterologous vaccination *via* mucosal routes may be advantageous for second-generation vaccines.

## Introduction

The currently licensed mRNA vaccines, administered parenterally, are highly effective at preventing severe disease and hospitalizations. However, control of the COVID-19 pandemic is continuously abrogated by ever-evolving variants of concern (VOCs). The mRNA vaccines have significantly reduced efficacy in preventing mild-to-moderate disease against VOCs as seen in the case of the delta and omicron variants (1–3). Further, there are reports of significant decreases in levels of pseudovirus neutralization from convalescent and vaccinated sera when compared to the ancestral strain (4). In addition, vaccinated individuals with breakthrough infections can still transmit virus to others (5). New FDA guidelines recommend that future booster shots target the spike (S) protein of new variants in addition to the ancestral Wuhan strain of SARS-CoV-2. Clinical trials testing a booster dose of Moderna’s BA.1-specific mRNA vaccine showed that serum from subjects vaccinated with an omicron-specific vaccine elicited higher neutralizing titers to BA.4/5 variants compared to those boosted with the currently authorized Wuhan-based vaccine (6). Although serum neutralizing antibodies were higher from the omicron-based vaccine, in a challenge study comparing the efficacy of these two vaccines in non-human primates (NHPs), similar levels of protection were observed after infection with the omicron variant (7). Further, recent data of individuals who received a fourth vaccination with either Wuhan- or omicron BA.4/5-based mRNA showed that BA.4/5 vaccination produced only a modest increase in neutralizing antibodies to BA.4/5 when compared to serum from individuals vaccinated with Wuhan-based vaccine (8, 9). It remains unclear if the inclusion of variant-specific genetic material *via* parenteral vaccination strategies will make a significant impact on the reduction of clinical disease or inhibit the spread the virus.

The high levels of circulating virus allow for continual rounds of viral evolution and the potential for new variants. An effective method of preventing future variants would be to block transmission. For respiratory pathogens such as SARS-CoV-2, the site of infection is the upper respiratory tract (URT), a part of the mucosal immune system. The URT is often the first line of defense against infection. It is believed that mucosal vaccinations will show improved efficacy and offer enhanced protection from SARS-CoV-2 vaccination by conferring immunity at the sites of infection (10, 11). A major component to mucosal immunity is the presence of dimeric and multimeric secretory IgA (S-IgA), which is secreted onto mucosal surfaces. Due to its valency, S-IgA is more neutralizing than monomeric antibodies (12, 13) and has been shown to play a critical role in preventing infection of respiratory pathogens (14–16). In addition, IgA from convalescent SARS-CoV-2 serum was found to be more neutralizing that IgG (17, 18). Further, it was found that mucosal immunity elicited by viral infection with the ancestral strain of SARS-CoV-2 provided protective immunity against omicron infection that was not observed after an intramuscular vaccination (19), highlighting the importance and potential power of second-generation, mucosally-delivered vaccines.

We have previously shown that oral and intranasal administration of rAd5-S-Wuhan in hamsters followed by breakthrough infection decreased transmission of SARS-CoV-2 to naïve hamsters (20) and protected hamsters from disease caused by the ancestral strain (21). A human phase I clinical trial showed that oral tablet immunization with VXA-CoV2-1, an adenoviral vector like the ones discussed below, expressing the Wuhan S and N, was able to generate mucosal IgA antibody responses that persisted for over one year and had higher surrogate neutralizing activity in mucosal samples than from convalescent subjects (22). Further, these mucosal antibodies were cross-reactive to other human coronaviruses like SARS-CoV-1, suggesting that mucosal immunity would be cross-reactive to future human coronaviruses, including emerging variants of SARS-CoV-2 (22). It remains unknown if mucosal vaccination would protect against disease caused by VOCs. In this study, we show the mucosal vaccination of Syrian hamsters with Wuhan or variant-based vaccines generates cross-reactive serum and mucosal antibodies and protects from challenge with the omicron and delta variants of SARS-CoV-2.

## Results

### Intranasal immunization with rAd5-S-Wuhan and rAd5-S-omicron antigens elicits cross-reactive serum antibody to VOCs

**Figure 1:**
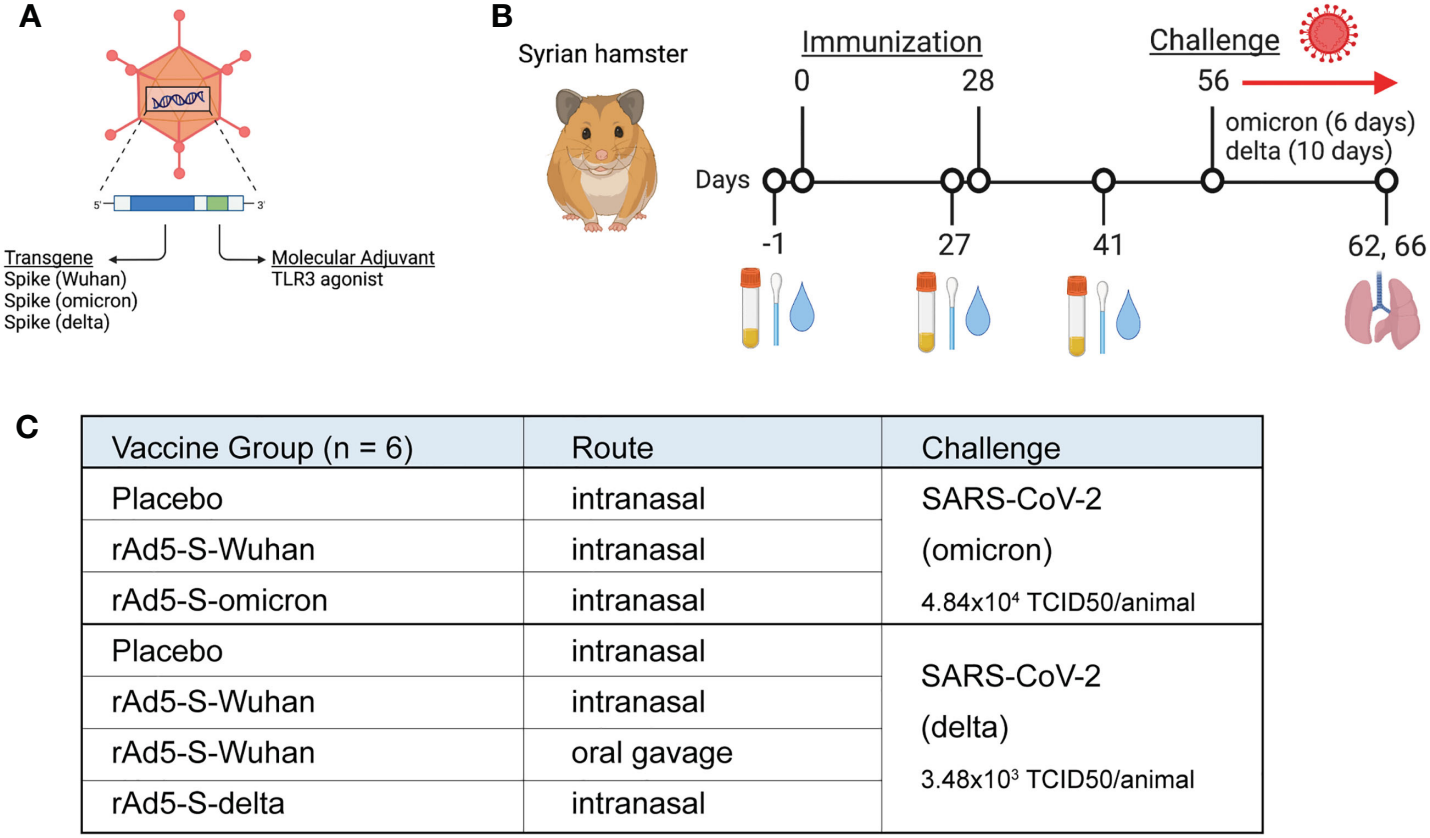
Study design for immunogenicity and efficacy of rAd5 vaccines against SARS-CoV-2 variants of concern. **(A)** Illustration of the rAd5 vector used in vaccination with the transgene (spike) and molecular adjuvant. **(B)** Schedule of vaccination, sample collection, and viral challenge. **(C)** Vaccination groups and administration routes in preparation for viral challenge. Figure was made with wwwbiorender.com.

To understand if mucosal vaccination with ancestral or variant-specific spike antigens could protect hamsters from disease caused by omicron and delta variants of concern (VOCs), we vaccinated hamsters with a non-replicating recombinant adenovirus type 5 (rAd5) that expresses a transgene of interest in the same gene cassette as a molecular adjuvant, a short nucleotide sequence that forms a hairpin RNA which acts as an innate immune antagonist within the same cell (**Figure 1A**) (23, 24). Hamsters (n=6/group) were immunized with 1x10^9^ infectious units (IU) of rAd5-S-omicron or rAd5-S-delta vaccines and compared to hamsters immunized with rAd5-S-Wuhan *via* the intranasal route in preparation for viral challenge (**Figures 1B, C**). Additionally, one group from the cohort with rAd5-S-delta received an oral administration of rAd5-S-Wuhan prior to challenge with delta virus (**Figure 1C**). Serum samples were collected at D-1 prior to primary vaccination, D27 (one day prior to boost), and D41 (two weeks post boost) and IgG and IgA were measured. Additionally, nasal washes and samples from oropharyngeal swabs were collected at these timepoints for the assessment of mucosal IgA (**Figure 1B**). Timepoints were collected before and after administration of boosting dose so that the effect of multiple vaccine doses on binding antibody levels and on antibody avidity could be assessed. At D56, one month post boost, animals were challenged with the homologous VOCs. The challenge doses were chosen based on previous Syrian hamster titration studies performed by Bioqual, Inc (Rockville, MD) where detectible levels of viral replication and/or disease could be observed. Samples from oropharyngeal swabs were collected each day after challenge and lung tissue was collected at the end of challenge for pathology and measurement of infectious viral load by TCID50. Infection with the omicron BA.1 variant causes only mild disease in hamsters and thus the challenge was ended after six days, as opposed to the 10 days observed with the delta variant challenge, so that pathology could be more easily assessed before the animals fully recovered.

**Figure 2:**
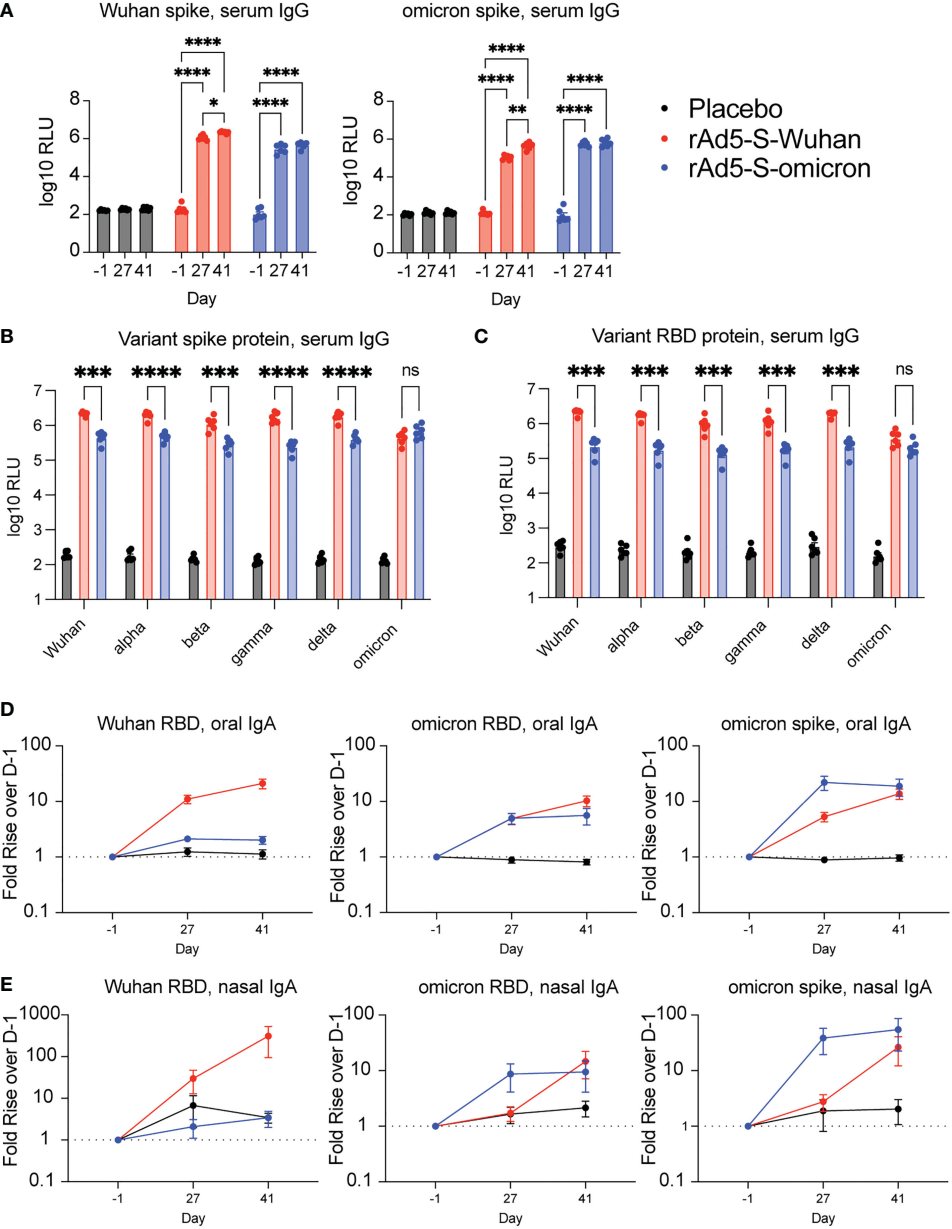
Serum and mucosal antibodies from Wuhan- and omicron-based vaccination. **(A)** Serum IgG from hamsters vaccinated with rAd5-S-Wuhan (red), rAd5-S-omicron (blue), or placebo (black) prior to vaccination (D-1), prior to boost (D27) and two weeks after boost (D41). **(B, C)** Levels of serum IgG to spike **(B)** and RBD **(C)** of SARS-CoV-2 VOC at D41 post prime vaccination. **(D)** IgA from oral swab eluates and **(E)** nasal washes at indicated times. Antibody signals were normalized to total IgA and plotted as fold rise of D-1. Mean and SEM plotted. 2-way ANOVA with a Geisser-Greenhouse correction and with Tukey’s multiple comparison test. *p < 0.05, **p < 0.01, ***p < 0.005, ****p < 0.0001. ns, not significant.

Serum was collected at indicated days post vaccination and IgG levels were quantified using the Meso Scale Discovery (MSD) platform utilizing electrochemiluminescent detection. As no spike-specific hamster antibodies exist for use as standards, values are reported as relative light units (RLU) and compared to either placebo-vaccinated or baseline (D-1) samples, rather than normalized to a standard curve. Vaccination with both rAd5-S-Wuhan and rAd5-S-omicron elicited cross reactive IgG to Wuhan, omicron and other VOC spike proteins after both prime and boost (**Figure 2A**). Further, a significant increase in binding antibodies to both Wuhan and omicron spike proteins was observed after boost vaccination with rAd5-S-Wuhan, but not with rAd5-S-omicron (p = 0.0119 and 0.0026, respectively), which reached its maximum level of binding by D27 (**Figure 2A**). Next, we tested whether the serum IgG at D41 were cross-reactive to other spike and RBD proteins of VOCs in addition to omicron. rAd5-S-Wuhan elicited cross-reactive IgG to variant spike and RBD proteins. In comparison, vaccination with rAd5-S-omicron still elicited cross-reactive IgG to Wuhan alpha, beta, gamma, and delta variant antigens tested, albeit at significantly reduced levels of binding antibody compared to rAd5-S-Wuhan (**Figures 2B, C**) (p < 0.005 – p <0.0001). Vaccination with rAd5-S-omicron and rAd5-S-Wuhan generated similar levels of binding IgG to omicron antigens (**Figures 2B, C**).

### Intranasal immunization with rAd5 expressing Wuhan and omicron antigens elicits cross-reactive oral and nasal IgA to VOCs

To determine if intranasal administration of rAd5-S vaccines elicited mucosal IgA, oral swab samples from D-1, D27, and D41 were analyzed for RBD and spike-specific IgA. Because experimental variability may be observed between swab samplings, specific IgA was first normalized to total IgA and expressed as fold rise over D-1 so that a trend could be observed. Both Wuhan and omicron vaccinations elicited increased RBD and spike-specific IgA compared to samples from pre-immune and placebo animals (**Figure 2D**). Similar to the serum samples, there were increases in binding following boost vaccination with rAd5-S-Wuhan, whereas boosting with rAd5-S-omicron did not increase the level of binding observed after prime vaccination (**Figure 2D**). Although not normalized to total IgA, raw values of spike-specific IgA mirrored these results (**Figure S1A**). As a second measure of mucosal IgA, nasal washes were collected at D-1, D27, and D41 of the study and evaluated for RBD and spike-specific IgA. Again, relative levels of spike specific IgA were determined by first normalizing to total IgA, then calculating the fold rise over D-1. Similar trends were seen when comparing antibody responses obtained from nasal washes of immunized animals, with all groups having increased omicron RBD and spike-specific IgA by D41 (**Figure 2E**, **Figure S1B**).

### Oral administration of rAd5-S-Wuhan elicits cross-reactive serum, oral, and nasal antibodies

In human phase I clinical trials using the same vaccine platform delivered to the ileum *via* enterically coated tablets, long lasting secretory IgA was observed in mucosal secretions (22). As a proxy for oral tablet delivery in humans, the second cohort of hamsters included a group that were vaccinated *via* oral gavage in parallel to groups with intranasal administration in preparation for challenge with the delta variant (**Figure 1C**). We sought to understand if the delivery of rAd5-S-Wuhan by oral gavage could generate cross-reactive antibodies in serum, nasal, and oral samples. Additionally, we wanted to see if immune responses from oral administration compared similarly to that of intranasal administration.

**Figure 3:**
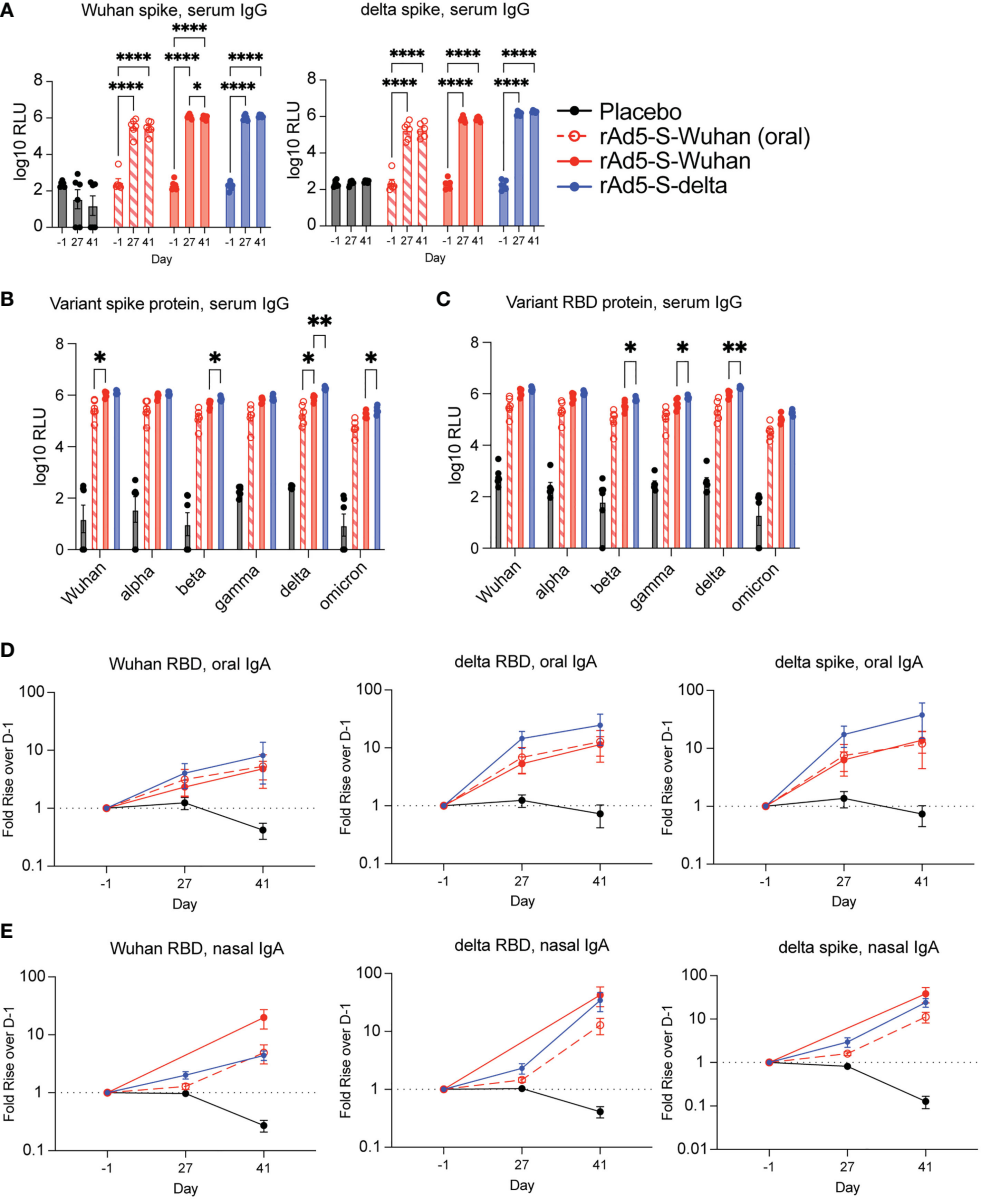
Serum and mucosal antibodies from Wuhan- and delta-based vaccination. **(A)** Serum IgG from hamsters vaccinated by oral gavage with rAd5-S-Wuhan (red – open shapes/dotted lines), intranasally with rAd5-S-Wuhan (red), rAd5-S-delta (blue), or placebo (black) prior to vaccination (D-1), prior to boost (D27) and two weeks after boost (D41). **(B, C)** Levels of serum IgG to spike **(B)** and RBD **(C)** of SARS-CoV-2 VOC at D41 post prime vaccination. **(D)** IgA from oral swab eluates and **(E)** nasal washes at indicated times. Antibody signals were normalized to total IgA and plotted as fold rise of D-1. Mean and SEM plotted. 2-way ANOVA with a Geisser-Greenhouse correction and with Tukey’s multiple comparison test. *p < 0.05, **p < 0.01, ****p < 0.0001.

Serum from vaccinated animals was analyzed for cross-reactive IgG against spike and RBD proteins. As this cohort of hamsters was vaccinated in preparation for challenge with the delta variant, Wuhan and delta antigens were assayed. Vaccination by oral gavage elicited significantly higher binding IgG to Wuhan and delta antigens over D-1 (p < 0.0001 at D27 and D41) (**Figure 3A**). In addition, intranasal vaccination with rAd5-S-Wuhan and rAd5-S-delta were able to generate cross-reactive IgG compared to baseline levels to both Wuhan and delta spike proteins (p < 0.001) (**Figure 3A**). Next, we tested whether the serum IgG at D41 from this cohort were cross-reactive to other spike and RBD proteins of VOCs in addition to delta. Intranasal administration of rAd5-S-Wuhan gave higher IgG responses to RBD and spike proteins than oral administration, although this difference was only significant in the case of Wuhan and delta spike proteins (p = 0.0472 and p = 0.0497) (**Figures 3B, C**). Intranasal administrations of rAd5-S-Wuhan and rAd5-S-delta elicited similar levels of serum IgG against spike and RBD, although vaccination with rAd5-S-delta performed significantly better against beta (p = 0.0423), delta (p = 0.0012), and omicron (p 0.0043) spike proteins, and beta (p = 0.0374), gamma (p = 0.0481), and delta RBD proteins (p = 0.0059) (**Figures 3B, C**). Oral administration of rAd5-S-Wuhan was not statistically compared to intranasal administration of rAd5-S-delta as both the antigen and route differed between the two groups.

In addition to quantifying the presence of serum IgG, we also observed IgA in mucosal secretions. Oropharyngeal swabs were tested for the presence of specific IgA as described in **Figure 2D**. At the timepoints tested, all vaccinated groups elicited higher IgA when compared to the pre-immune and placebo samples (**Figure 3D**). Although not normalized to total IgA, raw values of spike-specific IgA mirrored these results (**Figure S1A**). Lastly, we looked at nasal wash IgA as we were particularly interested to see if oral administration of rAd5 could elicit nasal IgA. By D41, all vaccinated groups tested showed cross-reactive antibodies in the nasal secretions (**Figure 3E**; **Figure S1D**). No specific or total nasal IgA was observed from the hamsters vaccinated intranasally with rAd5-S-Wuhan at D27, likely due to a technical error. Thus, this data point was excluded from analysis (**Figure 3E**; **Figure S1D**).

### Boost vaccination increases antibody avidity

**Figure 4:**
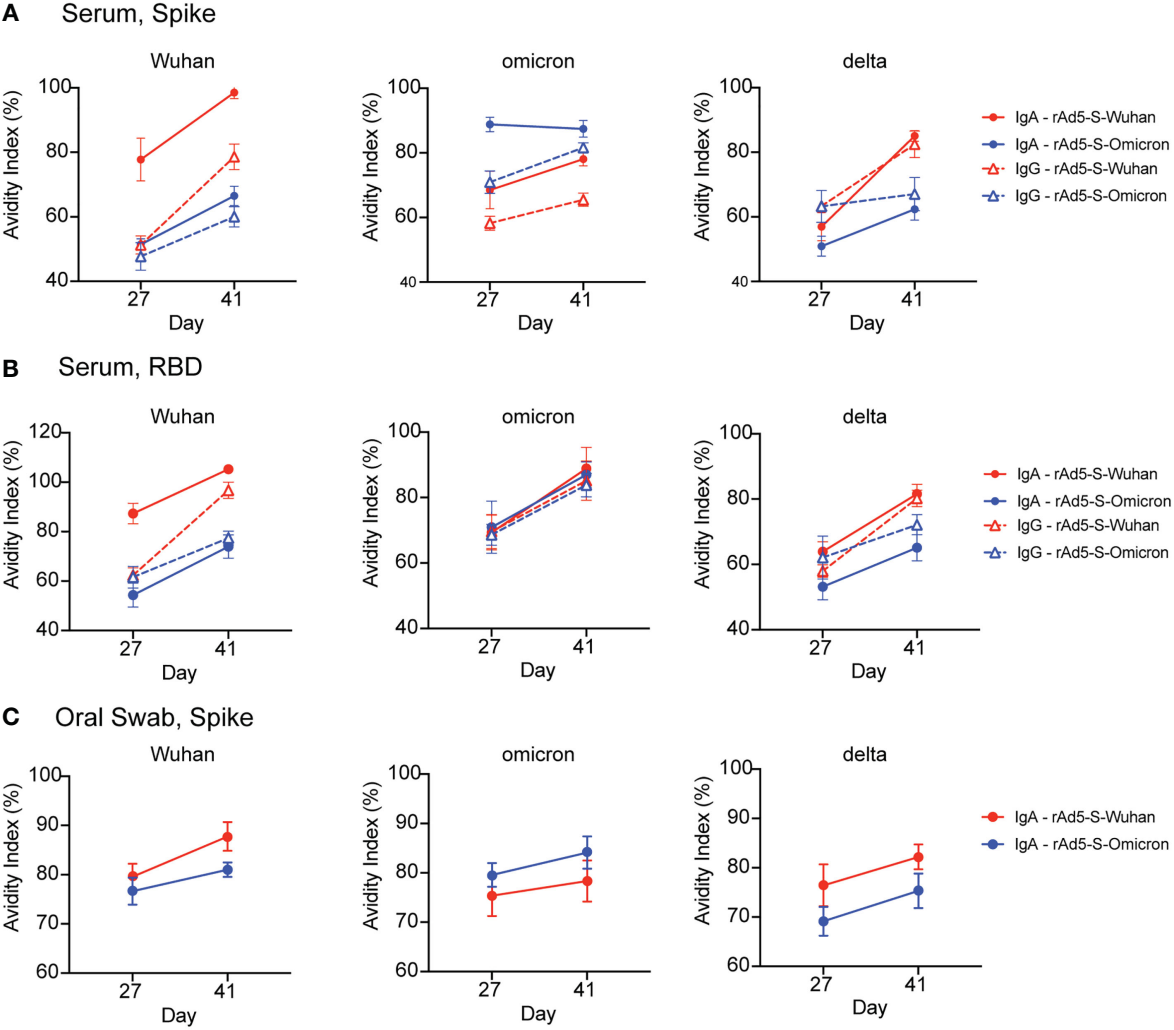
Antibody avidity following prime and boost vaccination. **(A, B)** Avidity index of serum IgA (solid lines, circles) and IgG (dashed lines/triangles) at D27 and D41 against SARS-CoV-2 spike **(A)** and RBD proteins **(B)**. **(C)** Avidity index of IgA eluted from oral swabs at D27 and D41 against SARS-CoV-2 spike protein. Mean and SEM plotted.

To determine if the antibodies generated by boost vaccination increased antibody specificity, we tested whether antibody avidity increased following boost vaccination by measuring the avidity index (25). In all iterations, vaccination with the matched antigen elicited the highest affinity antibodies against the homologous variant protein (**Figure 4**). The avidity index of both serum IgG and IgA and oral IgA to spike and/or RBD generally increased with boost. Interestingly, the IgA avidity index to omicron spike elicited by rAd5-S-omicron vaccination started high and stayed high (**Figure 4C**), whereas serum IgG increased in avidity with boost (**Figure 4A**). This phenomenon was only observed in the serum with respect to the spike protein as there was no superiority observed by antibody avidity towards omicron RBD with antibodies generated by both vaccinations giving equivalent avidity indexes (**Figure 4B**) and equal levels of binding antibody to omicron RBD (**Figure 2C**). Avidity of IgA from oral swabs also increased with boost, but this increase in avidity index was very slight (**Figure 4C**). Additionally, the avidity indexes of oral IgA were higher at D27 compared to the avidity index of serum IgG at D27 (**Figure 4**). This observation is in line with previous findings that secretory IgA is of higher avidity due to its valency. Lastly, as a comparator to other VOC proteins, the avidity index of antibodies generated after rAd5-S-Wuhan and rAd5-S-omicron vaccination were measured against delta spike and RBD proteins. In line with what was seen before, rAd5-S-Wuhan generated antibodies of the higher avidity when compared with the omicron vaccination, again indicating that Wuhan-based vaccination may elicit broadly cross-reactive antibodies across many VOCs, particularly in comparison to rAd5-S-omicron.

### Wuhan and omicron-based vaccines protect hamsters from disease caused by VOCs

**Figure 5:**
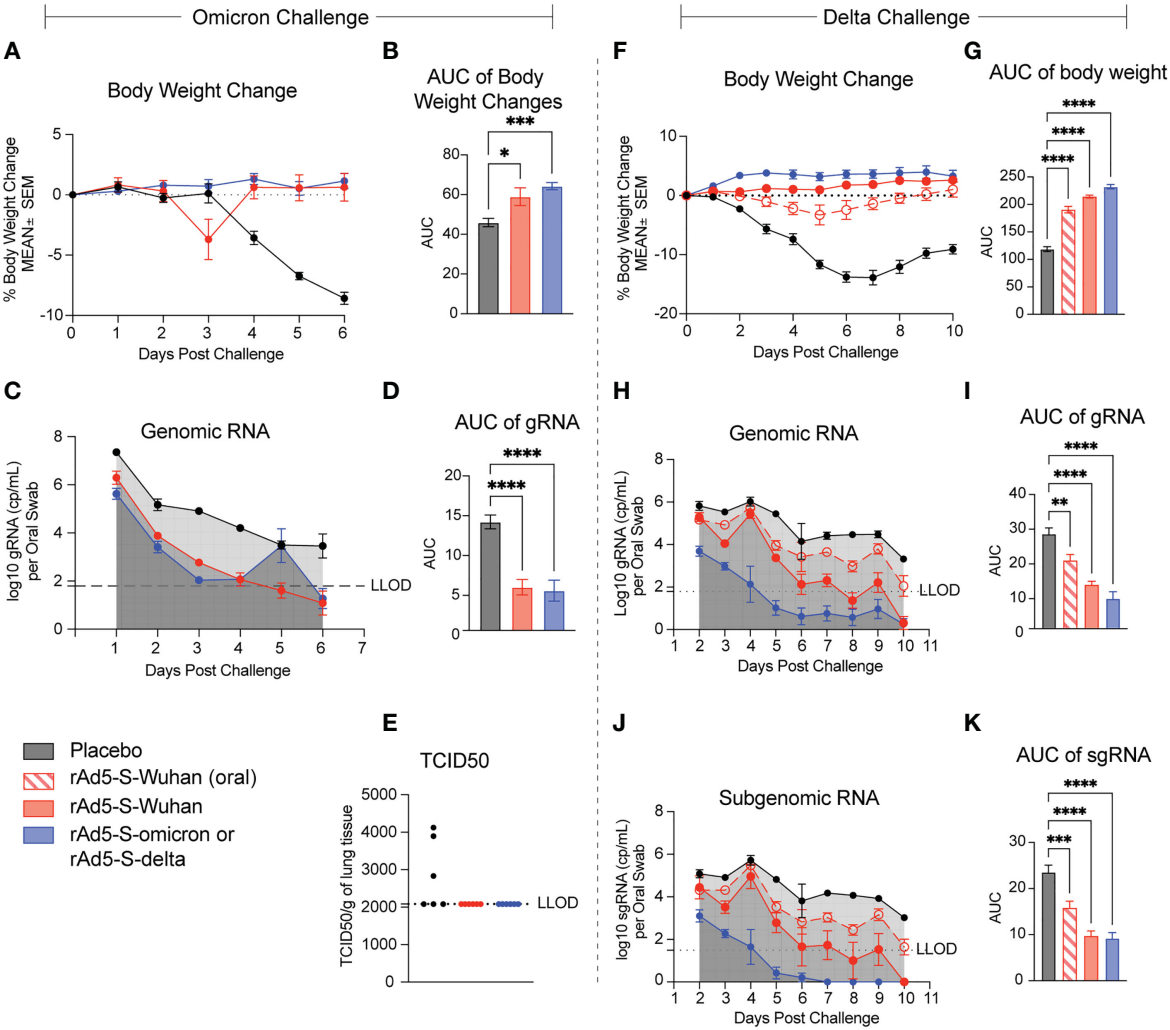
rAd5-S mediated protection of hamsters from disease caused by the omicron and delta variants. **(A)** Body weight changes and **(B)** Area under the curve (AUC) of hamsters vaccinated with rAd5-S-Wuhan (red), rAd-5-S-omicron (blue), and placebo (black) measured for six days after challenge. **(C)** Genomic (gRNA) detected from oral swabs of vaccinated hamsters and **(D)** AUC of the gRNA time course. **(E)** TCID50 of lung tissue at day six post infection. **(F)** Body weight changes and **(G)** AUC (One-way ANOVA) of hamsters vaccinated with oral rAd5-S-Wuhan (red – open shapes/dotted lines), rAd5-S-Wuhan (red, closed circles), rAd-5-S-delta (blue), and placebo (black) measured for 10 days after challenge. **(H)** Genomic (gRNA) detected from oral swabs of vaccinated hamsters and **(I)** AUC of the gRNA time course. **(J)** Subgenomic (sgRNA) and **(K)** AUC (one-way ANOVA) of the sgRNA time course. n=6, mean and SEM plotted. Ordinary one-way ANOVA with Dunnett’s multiple comparisons test. *p < 0.05, **p < 0.01, ***p < 0.005, ****p < 0.0001.

One month after vaccination series (D56), the omicron cohort of hamsters was challenged with 4.84x10^4^ TCID50 of the omicron BA.1 variant of SARS-CoV-2 and infection proceeded for six days. Both rAd5-S-Wuhan and rAd5-S-omicron protected hamsters from weight loss due to infection (p = 0.0186 and 0.0013 respectively) (**Figures 5A, B**). In addition, viral shedding, as measured by qRT-PCR of genomic RNA (gRNA) from oropharyngeal swab, was significantly reduced by both rAd5-S-Wuhan and rAd5-S-omicron compared to placebo (p = 0.0001 and p < 0.0001, respectively) (**Figures 5C, D**). At the end of the infection period, the lungs of hamsters were collected and TCID50 was determined. Three of the six hamsters in the placebo group had detectable levels of infectious virus in their lungs while no detectable virus was recovered from the lungs of vaccinated hamsters (**Figure 5E**).

The second cohort of hamsters was vaccinated with rAd5-S-Wuhan and rAd5-S-delta in preparation for challenge with the delta variant of SARS-CoV-2. Hamsters were infected with 3.48x10^3^ TCID50/animal and monitored for 10 days post challenge for weight loss and viral load. Animals vaccinated intranasally with rAd5-S-Wuhan and rAd5-S-delta experienced no weight loss compared to the placebo control. There was a small dip in body weight for hamsters vaccinated orally with rAd5-S-Wuhan, however all animals recovered quickly and all vaccine groups were significantly protected from weight loss compared to placebo (p=<0.0001 for all groups) (**Figures 5F, G**). Viral loads from oropharyngeal swabs were analyzed by qRT-PCR for subgenomic (sgRNA) and gRNA. Viral loads and viral replication were significantly reduced in the rAd5-S-Wuhan oral and intranasal groups with the greatest reduction of viral RNA levels in the homologous rAd5-S-delta vaccination group (p=0.0007, <0.0001, and <0.0001 respectively) (**Figures 5H–K**).

### Mucosal vaccination protects hamsters from lung pathology resulting from omicron and delta infection

**Figure 6:**
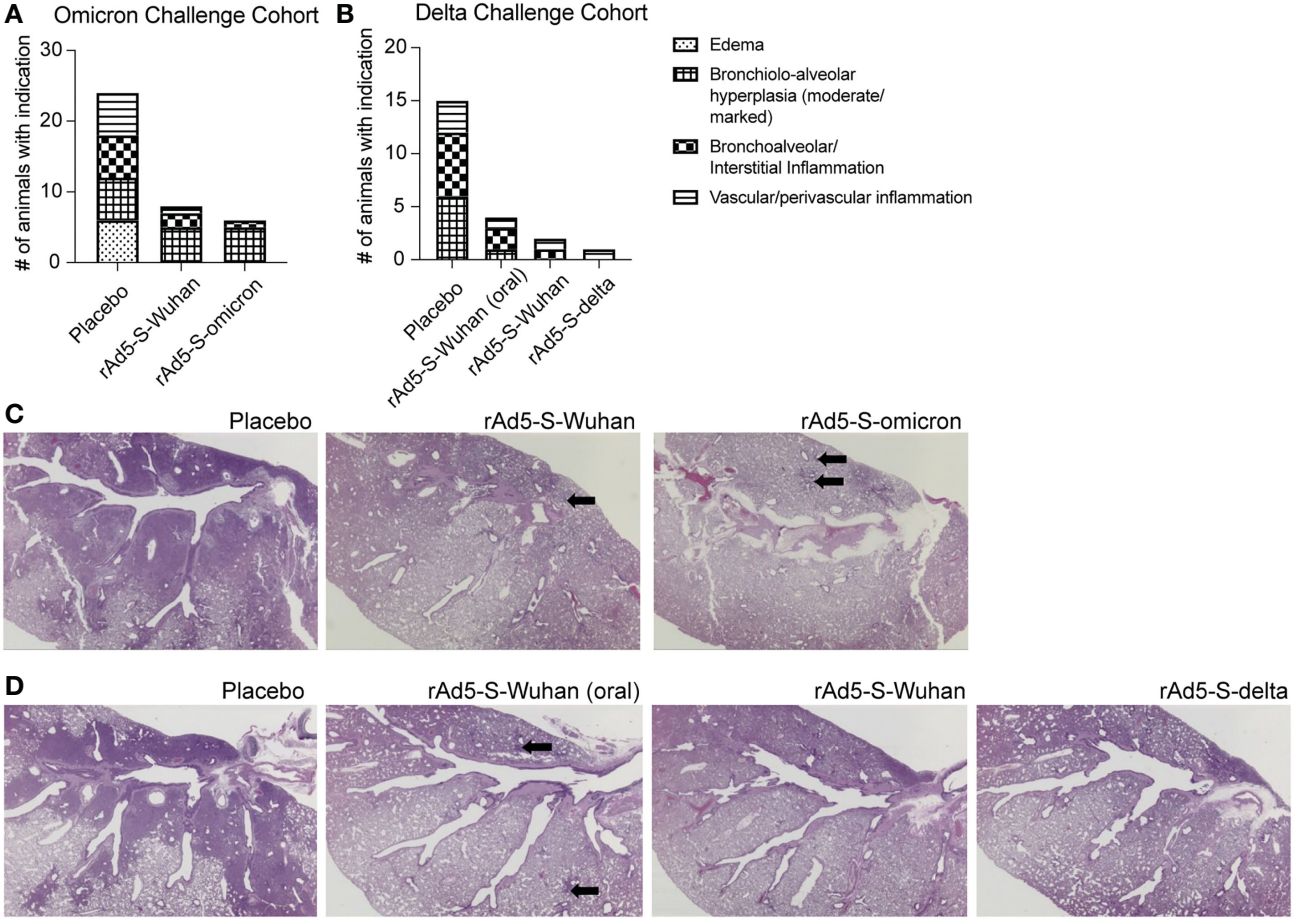
Lung pathology from placebo and rAd5-S vaccinated hamsters. **(A)** Number of animals with minimal/mild edema, moderate/marked bronchiolo-alveolar hyperplasia, minimal-moderate bronchoalveolar/interstitial inflammation, or minimal-mild vascular/perivascular inflammation from omicron cohort. **(B)** same as A, but from delta cohort. **(C, D)** Representative H&E of hamster lungs from the omicron cohort **(C)** and delta cohort **(D)**. Arrows indicate areas brochiolo-alveolar hyperplasia.

In addition to monitoring clinical symptoms of disease above, the lungs of infected animals were observed for signs of inflammation, as measured by bronchiolo-alveolar hyperplasia, and bronchoalveolar/interstitial and vascular/perivascular inflammation. Fewer animals from all vaccinated groups, as compared to placebo, showed signs of inflammation (**Figures 6A–D**). rAd5-S-delta was the most efficacious with the greatest decrease in severity of lung pathology compared to placebo animals in the cohort challenged with the delta variant. Bronchiolo-alveolar hyperplasia was observed in almost all animals challenged with either omicron or delta VOCs; however, severity was decreased compared to placebo treated animals. In the omicron cohort, there were no significant differences in efficaciousness between rAd5-Wuhan and r-Ad5-omicron. Overall, there were fewer animals with lung inflammation from the cohort infected with the delta variant than that of omicron. However, this is likely due to the timing of infection as by day 10 post infection with the delta variant, most of the animals had recovered in body weight (**Figures 5F, G**) whereas the cohort infected with the omicron variant had not yet recovered (**Figures 5A, B**).

## Discussion

In this study we demonstrate that mucosal delivery of recombinant adenovirus expressing the spike gene of Wuhan, delta, or omicron VOCs protected hamsters from disease caused by those variants. All vaccinated hamsters in this study developed serum IgG regardless of mucosal immunization route. Additionally, IgA was found in mucosal secretions from both the oral and intranasal cavity, again, regardless of mucosal immunization route.

It has been shown that immunization with a fourth dose of mRNA vaccine increases the serum neutralizing antibody titers in humans (4). Similarly, an increase in binding antibodies generated after boost vaccination with rAd5-S-Wuhan increased the level of binding antibodies to omicron RBD and spike proteins. We therefore wanted to determine if the avidity of antibodies generated by vaccination increased after an additional administration of vaccine. We found that both serum and mucosal antibodies increased in avidity to all spike variants tested following a booster dose.

When examining the ability of rAd5-vectored vaccines to protect against infection, immunization with the matched variant elicited the greatest reduction in clinical symptoms such as bodyweight loss and lung pathology. Viral load was also reduced in all vaccine groups compared to placebo. Genomic RNA is believed to represent primarily virion associated RNA, while subgenomic RNA is thought to represent active SARS-CoV-2 infection (26). In the omicron cohort, sgRNA was not observed, though it was concluded that a technical failure of primer binding prevented amplification, rather than a biological phenomenon. Although vaccination with the matched vaccine elicited the strongest protection from disease, significant levels of protection were still observed in the groups vaccinated with rAd5-S-Wuhan. Wuhan-based vaccination elicited significantly higher cross-reactive antibodies to most variant spike proteins when compared to omicron-based vaccination. This suggests that the immune response generated by mucosal vaccination of a Wuhan-based vaccine may be cross-protective to emerging VOCs. It remains unclear if boosting previously vaccinated individuals with a variant-specific vaccine would enhance variant-specific antibodies or rather boost previous Wuhan specific antibodies due to immune imprinting (27). It is likely that vaccination strategies that induce mucosal immunity, and thereby provide a more cross-reactive immune response, may be a favorable approach to future vaccination strategies rather than continuously modifying vaccines to currently circulating SARS-CoV-2 variants.

Since its emergence in 2019, SARS-CoV-2 has caused over 6.6 million deaths and over 650 million confirmed cases (28). The currently approved mRNA vaccines prevent severe disease, but do not protect against infection from ever evolving variants, particularly those of the omicron clade (29). Additionally, currently licensed mRNA vaccines yield reduced serum neutralization titers to the omicron variant as compared to the ancestral Wuhan strain, though the response is moderately improved after a third dose is administered (30–32). Further, immunity induced by parenteral vaccination does not effectively prevent transmission of SARS-CoV-2, allowing opportunities for continual variant evolution. New vaccination strategies are needed to curb the constant waves of new variant outbreaks. These strategies include the development of a pan-coronavirus vaccine that expresses the epitopes of several variants (33), a vaccine that targets more conserved epitopes from SARS-CoV-2 (34), or the induction of immunity at the site of infection, the respiratory mucosa, that can be cross-reactive to many variants of SARS-CoV-2. Inducing mucosal immunity would likely be an effective method to prevent transmission and block infection, largely due to the high neutralization activity of S-IgA (12, 13). In phase I clinical trials, we showed that vaccination *via* oral tablet vaccine induced mucosal SARS-CoV-2 specific IgA that possessed increased surrogate neutralizing activity (22). In pre-clinical animal models, we showed that our mucosal Wuhan-based vaccination construct protected against disease caused by the ancestral Wuhan strain of SARS-CoV-2 (20, 21). In a study from Harvell et al, it was observed that individuals with increased mucosal IgA had improved protection from breakthrough infection with the omicron (BA.1) variant of SARS-CoV-2 (19), illustrating the importance of inducing mucosal immunity to curb new waves of infection.

Despite showing cross-reactive IgA and IgG in our clinical and preclinical studies, it was unknown whether our rAd5-S vaccine would protect against VOCs such as omicron and delta variants. Other studies have shown that variant-specific vaccination, delivered parenterally, were effective at preventing disease in hamsters caused by delta and omicron variants, reducing the viral load in animal tissues after infection and protecting the animals from lung pathology (35, 36). Interestingly, a study by Halfmann et al. found that hamsters that had recovered from previous infection with the 614G/614D ancestral strain were better able to reduce BA.1 replication in both the nasal turbinates and lung tissue when compared with mRNA-vaccinated hamsters (37). This suggests that mucosal vaccination, even with a Wuhan-based vaccine, may provide increased protection against the omicron and delta VOCs.

A few limitations apply to our study. First, when used for human clinical trials (NCT04563702), this vaccine is formulated into enterically coated tablets that can be swallowed by subjects. These tablets can withstand the low pH of the stomach allowing for delivery of rAd5 to the ileum (23, 38–41). One limitation for this study is the use of intranasal vaccination in place of oral vaccinations. To mimic oral delivery, we included a group of hamsters in the delta cohort who received their vaccination *via* oral gavage. Oral gavage of small animals is difficult to deliver accurately (42) in comparison to intranasal delivery. However we were able to see comparable immunogenicity and protection from disease when the vaccine was delivered intranasally or by oral gavage, improving our confidence that use of intranasal delivery in our experimental models can aid our understanding of mucosal delivery of rAd5 generally (42). Another limitation is that each experiment was only performed once for both the omicron and delta cohorts, as the high cost of BSL-3 infections and the ever-evolving variants made an exact experimental repeat ill-favored. Although these challenge experiments were only performed once each, the observation that mucosal application of this rAd5-S is able to generate both serum and mucosal immune responses has been shown in other hamster studies (20, 21), NHP studies (43, 44), and studies from human clinical trials (22). Future experiments will be performed to further confirm our observations, and the data analyzed here will inform the experimental design of studies including those that utilize this mucosal rAd5-S vaccine to boost animals that have previously been immunized with mRNA vaccines in immunogenicity studies.

One concern in the use of adenovirus vectored vaccines is the development of anti-vector immunity. In a publication by O’Brien et al, intramuscular vaccination with an Ad5 vectored vaccine resulted in an >50 fold increase of anti-Ad5 antibodies (45). However, in a paper describing phase I human clinical trials for oral influenza vaccination, anti-Ad5 immunity did not increase after oral vaccination (40). Multiple groups, including ours, have shown that pre-existing immunity to Ad5 is not as problematic in mucosal-delivered Ad5 compared to what has been seen with parenteral vaccination (40, 46, 47). While anti-vector immunity was not measured in this study, we did observe a boosting effect on the antibody responses as well as an increase in avidity after boost vaccination, suggesting that a potential anti-vector response did not abrogate the boosting effect of mucosally delivered rAd5.

An orally delivered, thermo-stable, self-administered vaccine would have drastic impacts on public health and pandemic control, particularly in areas where cold-chain resources are limited. Further, generation of mucosal IgA through vaccination may have some substantial immunological benefits. Growing evidence suggests that mucosal IgA plays a key role in the prevention of infection, even when the prior exposure doesn’t match the circulating strains (19, 48). The nasal and oral mucosal surfaces are the first lines of defense against SARS-CoV-2 infection and local secretory IgA may do a better job of inhibiting infection than a systemic serum response. In a paper by Havervall et al, as little as 20 AU/mL of nasal IgA was effective at inhibiting viral infection in humans. Even if breakthrough infection occurs, mucosal vaccines may significantly reduce the spread to others, even to unvaccinated individuals (19). Several mucosal vaccine strategies have failed in humans, including intranasal rAd in two separate studies (49, 50). In contrast, we have previously shown that our clinical vaccine for SARS-CoV-2, VXA-CoV2-1, was well tolerated in humans when given as an oral tablet and generated cross-reactive mucosal antibodies and potent T-cell responses. In summary, the data presented here demonstrate that vaccination *via* oral and intranasal routes with both Wuhan and variant-matched vaccines, protected hamsters from disease caused by the omicron and delta variants of SARS-CoV-2. This technology is currently being evaluated in additional clinical trials and may offer a different, more complete approach to suppress pandemic SARS-CoV-2 worldwide compared to needle-based mRNA vaccination.

## Methods

### Animal model, study design and challenge

Male Golden Syrian hamsters (n=6) aged 6-8 weeks, were vaccinated according to the groupings in **Figure 1B** with a dose of 1x10^9^ infectious units (IU) diluted in 1xPBS in a total volume of 100 µL for intranasal vaccination (50 µL/nostril) and 1 mL total volume for oral gavage. Vaccinations occurred four weeks apart. Serum, nasal washes, and oral swabs were collected on day -1 (D1), day 27 (D27), and day 41 (D41) as described below. At D52, the animals were anesthetized by injecting 80 mg/kg ketamine and 5 mg/kg xylazine *via* intramuscular route in preparation for challenge. Animals were challenged with an appropriate dose *via* intranasal administration using a total volume of 100 μL per animal (50 μL/nostril), administered dropwise. SARS-CoV-2 delta variant (ATCC NR-56116 LOT#: 70047614) was dosed at 3.48x10^3^ TCID_50_ per hamster and SARS-CoV-2 omicron variant (ATCC NR-56486 LOT#: 70049695 was dosed at 4.8x10^4^ TCID_50_ per hamster as pre-determined in titration studies performed by Bioqual, Inc. Animals were monitored daily for any abnormal clinical observation and body weights were recorded. All in-life animal handling occurred at Bioqual, Inc (Rockville, MD).

### Virus generation

Adenovirus type 5 (rAd5) vaccines were generated based on the published spike sequence of SARS-CoV-2 Wuhan (Genbank Accession No. MN908947.3), delta (GISAID Accession No. EPI-ISL-2570775), and omicron (BA.1) (GISAID Accession No. EPI_ISL_6699769) variants. These sequences were inserted into a recombinant plasmid containing rAd5 sequence that is deleted in E1 and E3 genes. The respective transgenes were cloned into the E1 region that additionally contains a downstream molecular dsRNA adjuvant in the gene cassette and is expressed together with the transgene in the target cell. rAd5 particles were generated by transfection of transgene containing DNA into Expi293F cells (ThermoFisher Scientific) to generate rAd5 virions which were purified by CsCl density centrifugation (38, 51).

### Blood sample collection

Blood collection was performed one day prior to each vaccination (D-1, D27) and two weeks post final vaccination (D41). Prior to collection, animals were anesthetized with up to 5% isoflurane and blood was collected *via* the retro-orbital sinus vein. Samples were allowed to clot for 30 minutes – 1 hr at room temperature and centrifuged at 9300 x g for 10 minutes. Serum was stored at -80˚C.

### Nasal wash and oral swab collection

Hamster nasal wash samples were collected following anesthesia using isoflurane; a soft tipped catheter was used to flush 400 µL of 1X PBS into the nasal cavity and a collection device was placed under the opposite nostril to collect the fluid. The recovery yield was documented to be approximately 200 to 250 µL. Hamster oral swab samples for antibodies were collected using a sterile flocked swab (Copan Diagnostics FLOQSwabs 501CS01) by inserting into the mouth and swabbing both cheeks for at least 30 seconds. The swab was snap-frozen until elution in 200 µL 1X PBS, 0.25 M NaCl (Corning cat#21-031-CV, Acros Organics cat#327300025) and collected by centrifugation. Post-challenge swab samples were collected in cryovials containing 1 mL 1X PBS for qPCR viral load testing.

### Serum IgG responses against SARS-CoV-2 spike and RBD variants

Vaccine-induced serum IgG specific to SARS-CoV-2 spike and RBD variants was measured with MSD V-PLEX COVID-19 Serology kit panel 22 and 23 (MSD cat#K15563U, K15571U). Plates were coated, blocked, washed, and incubated with sample and detection antibody according to the manufacturer’s instructions. Serum samples were diluted at 1:4000 in Diluent-100 (MSD cat#R50AA). A goat anti-hamster IgG antibody (Invitrogen cat#*31115*) was sulfo-tagged with the MSD GOLD SULFO-TAG NHS-Ester Conjugation Pack (MSD cat#R31AA) and diluted to 1 µg/mL in Diluent-100. Plates were read on a Meso QuickPlex SQ 120 instrument (MSD) and sample values were reported as relative light units (RLUs). Data analysis was performed in GraphPad Prism (Version 9.4.1).

### Mucosal IgA responses against SARS-CoV-2 delta or omicron variant spike trimer and RBD

Vaccine-induced mucosal IgA specific to SARS-CoV-2 variant spike and RBD were measured with the Mesoscale Discovery (MSD) 4-spot U-PLEX Development Pack (MSD cat#K15229N). Spots were linked with anti-Syrian hamster IgA antibody (Brookwood Biomedical cat#sab3001a) biotinylated with the EZ-Link Sulfo-NHS-LC-Biotin kit (Thermo Fisher Scientific cat#A39257) and either biotinylated Wuhan, delta, or omicron variant SARS-CoV-2 spike trimer and RBD proteins (ACROBiosystems cat#SPD-C82E9, SPN-C82Ec, SPD-C82Ed, SPN-C82Ee, SPD-C82E4). Plates were coated, blocked, washed, and incubated with sample and detection antibody according to the manufacturer’s recommendations. Nasal samples were diluted at 1:15 and oral samples were diluted at 1:5 in Diluent-100 (MSD cat#R50AA). The anti-Syrian hamster IgA antibody was sulfo-tagged with the MSD GOLD SULFO-TAG NHS-Ester Conjugation Pack (MSD cat#R31AA) and diluted with Diluent-100 to 2 µg/mL for the nasal samples and 1 µg/mL for the oral samples. Plates were read on a Meso QuickPlex SQ 120 instrument (MSD). Samples were reported as relative light units (RLUs). Due to the variability in mucosal sampling, samples were normalized by total IgA and expressed as fold change was reported as vaccinated over unvaccinated sample. Data analysis was performed in GraphPad Prism (Version 9.4.1).

### Avidity assay

Serum and mucosal antibody avidity was determined by MSD using either MSD V-PLEX COVID-19 Serology kit panel 25 (MSD cat# K15583U-2) or U-PLEX Development Pack (MSD cat#K15229N). U-plex spots were linked with biotinylated Wuhan, delta, or omicron variant SARS-CoV-2 spike trimer and RBD proteins (ACROBiosystems cat#SPD-C82E9, SPN-C82Ec, SPN-C82Ee, SPD-C82E4). Plates were coated, blocked, washed, and incubated with sample and detection antibody according to the manufacturer’s recommendations and as described above with the exception that after incubation of sample, an additional 10-minute incubation of 3M urea in 1X PBS + 0.05% Tween-20 was performed. Avidity was calculated as (RLU_urea_)/(RLU_PBST_)*100.

### TCID50 assay

Tissue sections were homogenized in 0.5 mL cold medium (DMEM + 10% FBS + 0.05 mg/mL gentamicin) for and centrifuged (2000 xg, 4°C, 10 minutes) to remove debris and supernatants collected. Clear flat-bottom 96-well culture microplates (BD Falcon Cat. #: 353072) were seeded with Vero TMPRSS2 cells at 2.5x10^4^ cells per well in growth media (DMEM + 10% FBS + 1% Penicillin/Streptomycin + 10 µg/mL Puromycin) and incubated at 37°C, 5% CO2 until 80-100% confluent. A 10-fold dilution series of processed tissue sample was plated in growth media (DMEM + 2% FBS + 1% Penicillin/Streptomycin + 10 µg/mL Puromycin). Plates were incubated at 37°C, 5% CO_2_ for 4 days. After incubation, the presence of cytopathic effects (CPE) was plated, and the TCID50 value per mL was calculated using the Read-Muench formula based on the tissue section weight, homogenization volume, and sample volume used in the assay assigned a calculated TCID_50_ titer per gram of tissue. Data analysis was performed in GraphPad Prism (Version 9.4.1).

### Quantitative RT-PCR assay for SARS-CoV-2 oral swabs

Post-challenge oral swabs were analyzed at the DUKE Human Vaccine Center IVQAC. The assay for SARS-CoV-2 quantitative Polymerase Chain Reaction (qPCR) detects total RNA using the WHO primer/probe set E_Sarbeco (Charité/Berlin). A QIAsymphony DSP, automated sample preparation platform along with a virus/pathogen DSP midi kit, were used to extract viral RNA from 800 μL of sample. A reverse primer specific to the envelope gene of SARS-CoV-2 (5′-ATA TTG CAG CAG TAC GCA CAC A-3′) was annealed and then reverse transcribed into cDNA using SuperScript™ III Reverse Transcriptase with RNase Out. The resulting cDNA was treated with RNase H (Thermo Fisher Scientific) and then added to a custom 4x TaqMan™ Gene Expression Master Mix containing primers and a fluorescently labeled hydrolysis probe specific for the envelope gene of SARS-CoV-2 (forward primer 5′-ACA GGT ACG TTA ATA GTT AAT AGC GT-3′, reverse primer 5′-ATA TTG CAG CAG TAC GCA CAC A-3′, probe 5′-6FAM/AC ACT AGC C/ZENA TCC TTA CTG CGC TTC G/IABkFQ-3′). SARS-CoV-2 RNA copies per reaction were interpolated using quantification cycle data and a serial dilution of a highly characterized custom DNA plasmid containing the SARS-CoV-2 envelope gene sequence. Mean RNA copies per milliliter were then calculated by applying the assay dilution factor with a limit of detection (LOD) approximately 62 RNA copies per mL of sample.

SARS-CoV-2 N gene subgenomic mRNA was measured by a one-step RT-qPCR. To generate standard curves, a SARS-CoV-2 E gene sgRNA sequence, including the 5′UTR leader sequence, transcriptional regulatory sequence, and the first 228 bp of E gene, was cloned into a pcDNA3.1 plasmid. For generating SARS-CoV-2 N gene sgRNA, the E gene was replaced with the first 227 bp of N gene. The respectively pcDNA3.1 plasmids were linearized, transcribed using MEGAscript T7 Transcription Kit, and purified with MEGAclear Transcription Clean-Up Kit. The purified RNA products were quantified on Nanodrop, serial diluted, and aliquoted as E sgRNA or N sgRNA standards. RNA extracted from samples or standards were then measured in Taqman custom gene expression assays using TaqMan Fast Virus 1-Step Master Mix and custom primers/probes targeting the E gene sgRNA (F primer: 5′ CGATCTCTTGTAGATCTGTTCTCE 3′; R primer: 5′ ATATTGCAGCAGTACGCACACA 3′; probe: 5′ FAM-ACACTAGCCATCCTTACTGCGCTTCG-BHQ1 3′) or the N gene sgRNA (F primer: 5′ CGATCTCTTGTAGATCTGTTCTC 3′; R primer: 5′ GGTGAACCAAGACGCAGTAT 3′; probe: 5′ FAM-TAACCAGAATGGAGAACGCAGTG GG-BHQ1 3′). Standard curves were used to calculate E sgRNA in copies per mL; the limit of detections (LOD) for N sgRNA assays were approximately 31 copies per mL of sample.

### Pathology

At necropsy, the left lung of each animal was collected and placed in 10% neutral buffered formalin. Tissue sections were trimmed and processed to hematoxylin and eosin (H&E) stained slides and examined by a board-certified pathologist at Experimental Pathology laboratories, Inc. (EPL) (Sterling, VA). Findings were graded from one to five (1=minimal, 2=mild, 3=moderate, 4=marked, 5=severe).

## Data availability statement

The raw data supporting the conclusions of this article will be made available by the authors, without undue reservation.

## Ethics statement

The animal study was reviewed and approved by BIOQUAL Institutional Animal Care and Use Committee, BIOQUAL, Inc.

## Author contributions

MB and ST conceptualized and designed all experiments. ED and LS generated the vaccine material. CM, MB, and AM performed the immunological experiments in **Figures 2**–**4** and **S1**. MB and CM analyzed the data in **Figures 2**–**4** and **S1**. MB and staff at Bioqual, Inc. analyzed the data in **Figures 5**–**6**. MB and wrote the original draft with the support of ST. All authors contributed to the article and approved the submitted version.

## Supplementary material

Supplementary Figure 1 Raw RLU from spike-specific mucosal IgA. **(A)** RLU values of RBD or spike specific IgA from oral swabs from placebo (black), rAd5-S-Wuhan (red), or rAd5-S-omicron (blue) vaccinated hamsters. **(B)**. RLU values of RBD or spike specific IgA from oral swabs from placebo (black), oral administration of rAd5-S-Wuhan (red, open circles), intranasal administration of rAd5-S-Wuhan (red, closed circles), or rAd5-S-delta (blue) vaccinated hamsters.n=6, mean and SEM plotted.
