# Supplementary figures and images for: Mucosal immunization with Ad5-based vaccines protects Syrian hamsters from challenge with omicron and delta variants of SARS-CoV-2
Source: Front Immunol. 2023 Feb 22;14:1086035. doi: 10.3389/fimmu.2023.1086035 (PMC9992185; doi:10.3389/fimmu.2023.1086035)

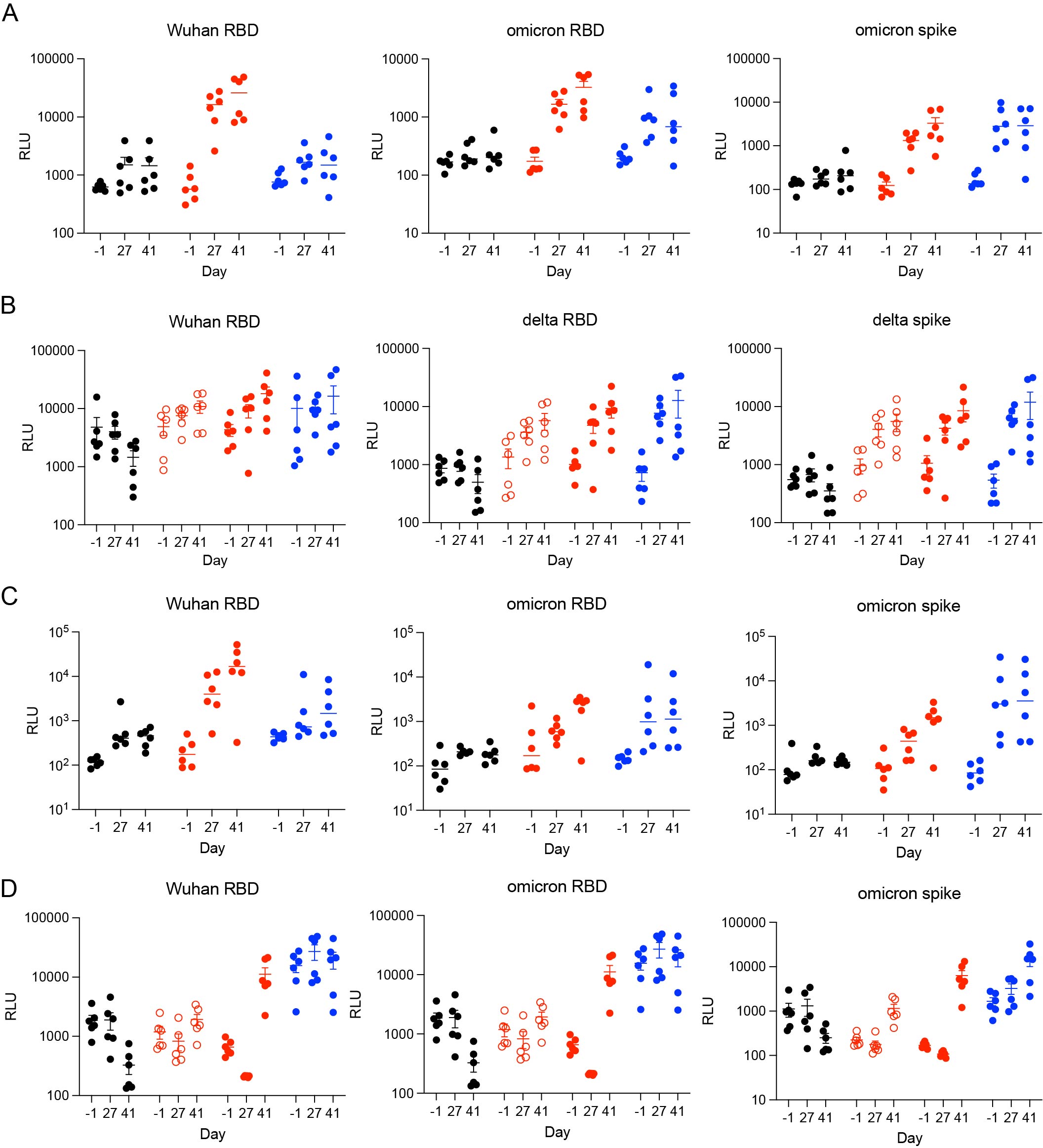

Supplement: Supplementary Figure 1 — Raw RLU from spike-specific mucosal IgA. (A) RLU values of RBD or spike specific IgA from oral swabs from placebo (black), rAd5-S-Wuhan (red), or rAd5-S-omicron (blue) vaccinated hamsters. (B). RLU values of RBD or spike specific IgA from oral swabs from placebo (black), oral administration of rAd5-S-Wuhan (red, open circles), intranasal administration of rAd5-S-Wuhan (red, closed circles), or rAd5-S-delta (blue) vaccinated hamsters.n=6, mean and SEM plotted. [file Image_1.jpg]
